# Treatment of Late Stage Disease in a Model of Arenaviral Hemorrhagic Fever: T-705 Efficacy and Reduced Toxicity Suggests an Alternative to Ribavirin

**DOI:** 10.1371/journal.pone.0003725

**Published:** 2008-11-14

**Authors:** Brian B. Gowen, Donald F. Smee, Min-Hui Wong, Jeffery O. Hall, Kie-Hoon Jung, Kevin W. Bailey, John R. Stevens, Yousuke Furuta, John D. Morrey

**Affiliations:** 1 Institute for Antiviral Research, Logan, Utah, United States of America; 2 Department of Animal, Dairy, and Veterinary Sciences, Utah State University, Logan, Utah, United States of America; 3 Department of Mathematics and Statistics, Utah State University, Logan, Utah, United States of America; 4 Research Laboratories, Toyama Chemical Company, Ltd., Toyama, Japan; Institut Pasteur Korea, Republic of Korea

## Abstract

A growing number of arenaviruses are known to cause viral hemorrhagic fever (HF), a severe and life-threatening syndrome characterized by fever, malaise, and increased vascular permeability. Ribavirin, the only licensed antiviral indicated for the treatment of certain arenaviral HFs, has had mixed success and significant toxicity. Since severe arenaviral infections initially do not present with distinguishing symptoms and are difficult to clinically diagnose at early stages, it is of utmost importance to identify antiviral therapies effective at later stages of infection. We have previously reported that T-705, a substituted pyrazine derivative currently under development as an anti-influenza drug, is highly active in hamsters infected with Pichinde virus when the drug is administered orally early during the course of infection. Here we demonstrate that T-705 offers significant protection against this lethal arenaviral infection in hamsters when treatment is begun after the animals are ill and the day before the animals begin to succumb to disease. Importantly, this coincides with the time when peak viral loads are present in most organs and considerable tissue damage is evident. We also show that T-705 is as effective as, and less toxic than, ribavirin, as infected T-705-treated hamsters on average maintain their weight better and recover more rapidly than animals treated with ribavirin. Further, there was no added benefit to combination therapy with T-705 and ribavirin. Finally, pharmacokinetic data indicate that plasma T-705 levels following oral administration are markedly reduced during the latter stages of disease, and may contribute to the reduced efficacy seen when treatment is withheld until day 7 of infection. Our findings support further pre-clinical development of T-705 for the treatment of severe arenaviral infections.

## Introduction

The number of highly pathogenic arenaviruses that can cause a severe syndrome known as viral hemorrhagic fever (HF) has recently expanded with the addition of Chapare virus to the growing list that includes Junin, Guanarito, Machupo, Sabia, and Lassa viruses [1]. Humans are believed to contract arenaviral HFs through contact with excreta from persistently infected rodents that serve as reservoirs for these viruses [2]. As a consequence of the growing human population and its encroachment into previously unpopulated areas, there is an increased opportunity for co-habitation between humans and the rodents that harbor highly pathogenic arenaviruses. The increasing frequency of human activity in areas where there is potential for inhalation of aerosolized infected rodent excrement will almost certainly lead to the discovery of additional arenaviral human pathogens. Importantly, the fact that these viruses are all infectious via aerosol exposure increases the potential for their use as bioterror agents [3].

Studies investigating ribavirin usage for the treatment of Lassa fever (LF), and Argentine and Bolivian HFs, suggest that it may have some utility [4], [5], [6]. However, in addition to concerns regarding toxicity and lack of specificity, the most compelling evidence for ribavirin application is based on a single human study employing a historical control group, wherein ribavirin therapy produced mixed results in treating severe LF cases [6]. When given within 6 days of disease onset, intravenous ribavirin dramatically reduced mortality in patients considered to be at greatest risk, whereas those starting therapy after that window of treatment initiation did not fare as well. Ribavirin is the only drug that has been recommended for use in treating arenaviral HFs under emergency provisions [7]; however, it has not been approved by the FDA for this indication. The idea of approving ribavirin, a drug known to induce hemolytic anemia, as a standard treatment for arenaviral HFs, where blood loss can be associated with severe disease, must be carefully considered.

There is an important need for safe and effective antivirals with broad-spectrum activity encompassing multiple RNA virus families. The demonstrated activity of T-705 (6-fluoro-3-hydroxy-2-pyrazinecarboxamide), a novel pyrazine derivative, in successfully treating in vitro and in vivo viral infections caused by a growing number of RNA viruses including several arenaviruses [8], bunyaviruses [8], flaviviruses [9](J. Julander, unpublished data), picornaviruses [10], orthomyxoviruses [10], [11], [12], paramyxoviruses [10], and togaviruses (J. Julander, unpublished data), is remarkable. Although much work remains to be done, the lack of toxicity and apparent broad-spectrum activities of T-705 are very encouraging in that T-705 may provide therapeutic solutions for multiple viral diseases. T-705 is currently in clinical development for the treatment of influenza virus infections in Japan and the USA [13]. Approval for use in treating seasonal and highly pathogenic avian influenza would facilitate approval for other indications, as PK, safety testing, and dosing data will be available from the clinical trials.

We have recently reported that T-705, recognized primarily for its remarkable activity against a variety of influenza virus infections in mice [10], [11], [12], was highly active in cell culture against several arenaviruses (Tacaribe, Junin, and Pichinde), with considerably less toxicity and superior selectivity indices compared to ribavirin [8]. More importantly, T-705 was also efficacious in vivo in the Pichinde virus (PICV) hamster infection model of arenaviral HF [14]. As biosafety level 4 (BSL-4) maximum containment facilities required for working with arenaviral HF agents are not readily available to most researchers, the less biohazardous PICV infection model (BSL-2) has facilitated early stage preclinical evaluation of T-705 and other drugs [8], [15], [16], [17]. As the previous studies were directed at establishing proof-of-concept, T-705 treatments were initiated within 72 h after challenge with a lethal dose of PICV [8], consistent with prophylactic use in the event accidental laboratory infection where therapeutic intervention could be started early. Since there is a clear need for antiviral therapies that are effective during the later stages of severe arenaviral infections, here we report the use of T-705 for treating advanced PICV disease in hamsters.

## Materials and Methods

### Animals and virus

Female 7–8 week-old Syrian hamsters were obtained from Charles River Laboratories (Wilmington, MA). Animals were acclimated to the Laboratory Animal Research Facility at Utah State University for 6–7 days prior to use. Experimental procedures involving hamsters complied with USDA guidelines and were approved by the Utah State University Institutional Animal Care and Use Committee.

PICV, strain An 4763, was provided by Dr. David Gangemi (Clemson University, Clemson, South Carolina). The viral stock was prepared following a single passage through hamsters and was the same as that used in previously published reports [8], [15], [16]. Briefly, the stock was generated by homogenization of pooled livers harvested from infected hamsters. Homogenates were clarified by high-speed centrifugation, aliquoted, and stored at −80°C. The stock virus concentration was ∼1×10^8^ plaque-forming units per ml. Virus was prepared for inoculation in minimal essential medium and given bilaterally in two intraperitoneal (i.p.) injections of 0.1 mL.

### Antiviral drugs

T-705 was kindly provided by the Toyama Chemical Company, Ltd. (Tokyo, Japan) and suspended in 0.4% carboxymethylcellulose (CMC). Ribavirin was supplied by ICN Pharmaceuticals, Inc. (Costa Mesa, CA) and was dissolved in sterile saline solution. All treatments were given by oral gavage (p.o.).

### Hamster PICV challenge and antiviral treatment studies

For all experiments, hamsters were weighed prior to the start of experiments and sorted into groups of 15–25 hamsters so that mean group weights across the entire experiment varied by less than 5%. Hamsters were challenged i.p. with ∼2 plaque-forming units of PICV. Groups of hamsters received treatments as specified in the figure legends or Table 1. Five animals from each treatment group were sacrificed on day 7 of infection to measure viral loads and serum alanine aminotransferase (ALT) activity. Serum was collected for assaying systemic viral burden and ALT activity. Livers were harvested and homogenized for viral titer determination as described below. The remaining 10–20 animals in each group were held 21–28 days for observation. Several sham-infected animals were included in each experiment as normal controls in order to establish reference values for all tested parameters. In several experiments, mean group or individual hamster weights were determined periodically through the duration of the study to assess illness, recovery, and general health status.

**Table 1:** Effect of a seven-day, twice daily, oral T-705 and ribavirin combination treatment initiated on day 5 of PICV infection in hamsters.

					Disease parameter^b^		
			Mean weight		Mean virus titer^c^±SD		
Treatment	Dosage/kg/day	Surv/total	Day 28 (g)	MDD^a^±SD	Liver	Serum	ALT^d^±SD
T-705+Ribavirin	50 mg+30 mg	5/10	142	24.0±3.5***	5.8±1.1 (100)**	<3.7±1.5 (100)**	30±21**
T-705+Ribavirin	10 mg+30 mg	5/10	113	21.2±2.2***	6.8±0.6 (100)**	5.0±0.9 (100)**	27±29**
T-705+Ribavirin	0 mg+30 mg	6/10*	104	22.0±3.6***	6.9±0.9 (100)**	4.8±1.2 (100)**	23±29**
T-705+Ribavirin	50 mg+6 mg	8/10**	131	14.5±9.2	6.7±1.2 (100)**	<3.9±1.3 (80)**	239±226**
T-705+Ribavirin	10 mg+6 mg	2/10	108	12.8±5.7*	6.8±1.3 (100)**	<5.1±1.8 (100)**	420±486 **
T-705+Ribavirin	0 mg+6 mg	5/10	96	11.6±5.3	8.1±0.5 (100)*	>7.3±0.9 (100)	794±441**
T-705+Ribavirin	50 mg+0 mg	3/10	148	14.1±5.1**	>8.4±0.8 (100)	>6.6±1.6 (100)*	1460±1308
T-705+Ribavirin	10 mg+0 mg	1/10	142	8.9±0.9	>8.8±1.2 (100)	>8.2±0.4 (100)	2678±1833
T-705+Ribavirin	0 mg+0 mg	4/20	120	8.8±1.6	>9.2±0.4 (100)	>8.2±0.4 (100)	2517±605
T-705	100 mg	10/10***	134	>28	5.6±0.8 (100)**	<3.6±1.8 (80)**	256±252**
Ribavirin	60 mg	10/10***	113	>28	6.3±0.9 (100)**	<4.5±1.7 (100)**	16±7**
Sham-infected	-	4/4	168	>28	<2.8 (0)	<1.8 (0)	33±10

a Mean day of death of hamsters dying prior to day 28.

b Determined on day 7 of infection; 5 hamsters per treatment group.

c Log_10_ cell culture 50% infectious dose (CCID_50_)/g of liver or ml of serum. Percentage of animals presenting with detectable virus levels are indicated in parenthesis.

d Alanine aminotransferase; measured in international units per liter.

* P<0.05; ^**^P<0.01; ^***^P<0.001 compared to 0.4% CMC placebo-treated hamsters.

For the study of temporal analysis of PICV tissue viral titers and other disease parameters hamsters in each group were challenged with ∼2 PFU of PCV. Groups of PICV-challenged hamsters were sacrificed daily over the course of 10 days. Serum, liver, spleen, lung, kidney, intestine and brain tissue were collected from each animal at the time of sacrifice. Serum was collected for assaying ALT, aspartate aminotransferase (AST) and type I interferon (IFN) amounts, and virus titers were determined for serum and clarified tissue homogenates as described below.

### Determination of infectious virus in serum and tissues

Virus titers were assayed using an infectious cell culture assay as previously described [17]. Briefly, a specific volume of tissue homogenate or serum was serially diluted and added to triplicate wells of BS-C-1 (African green monkey kidney) cell monolayers in 96-well microplates. The viral cytopathic effect (CPE) was determined 7–8 days post-virus inoculation and the 50% endpoints were calculated as described [18]. The assay detection range was 2.75–9.5 log_10_ cell culture 50% infectious doses (CCID_50_)/g of tissue or 1.75–8.5 CCID_50_/ml of serum. For statistical analysis of samples presenting with undetectable tissue or serum virus, a value of 2.75 or 1.75 log_10_ was assigned, respectively. In cases where virus exceeded the detection range, a value of 9.5 or 8.5 log_10_ was assigned. Thus, where samples are shown to be at the outer limits of detection, group mean viral loads may be an over- or under-representation of the actual burden.

### Serum ALT and AST determinations

Serum ALT concentrations were measured using a kinetic assay employing the ALT (SGPT) Reagent Set from Pointe Scientific, Inc. (Lincoln Park, MI). Serum AST concentration were measured using the AST Reagent Set from Teco Diagnostics (Anaheim, CA), which is a colorimetric endpoint assay. Assays were performed in accordance to the manufacturer's recommendations, adjusting the reagent volumes for both assays for analysis of samples in 96-well microplate format.

### Hamster type I IFN bioassay

Induction of type I IFN was determined using a bioassay adapted for the hamster system from previously described methods [19]. Briefly, baby hamster kidney (BHK-21) cells seeded in 96-well half-growth area, clear bottom, white opaque polystyrene microplates were pre-incubated with serially diluted serum samples. After a 6–8 hour pre-incubation period, a predetermined amount of the Indiana strain of vesicular stomatitis virus (VSV) was added to each well, except for the uninfected cell controls, and the ability of the samples to inhibit cell death (cytopathic effect; CPE) was evaluated. Upon reaching >80% CPE by visual examination (18–24 h post-infection), cell viability across the entire plate was measured by assaying for the presence of ATP using the Cell-Titer Glo® system from Promega (Madison, WI). Luminescence was read on a LB960 Centro luminometer from Berthold Technologies (Oak Ridge, TN). Type I IFN titers were based on a 50% reduction of viral cytotoxicity and were determined by regression analysis.

### Analysis of plasma T-705 concentrations following oral treatment of infected and uninfected hamsters

Groups of 3 hamsters each were either inoculated i.p. with ∼5 PFU of PICV or sham-infected. On day 7 of the infection, animals were given 50 mg/kg T-705 p.o. and animals were sacrificed for plasma collection at 0.25, 0.5, 1, 2 and 4 h following treatment for pharmacokinetic (PK) analysis of T-705 and its primary (dead-end) metabolite, T-705M1 (6-fluoro-3,5-dihydroxy-2-pyrazinecarboxamide). Plasma was deproteinized by the addition of equal volume of acetonitrile/methanol (1∶1) and the clarified solution was evaporated prior to reconstitution in the mobile phase for high-pressure liquid chromatography (HPLC) analysis.

Separation and quantitation of T-705 and T-705M1 (devoid of antiviral activity) were performed by HPLC. Briefly, 100-µl samples were analyzed using a Varian HPLC system (Varian Chromatography Systems, Woburn, MA) fitted with a Thermo Scientific 4.6 mm×25 cm Hypersil ODS C-18 reverse phase column using an isocratic buffer comprised of 1% acetonitrile in 100 mM triethylammonium phosphate (prepared with phosphoric acid titrated to pH 6.5 with triethylamine) [20]. An optical density tracing during the separation was made at 360 nm using an integrator (Shimadzu Instruments, Columbia, MD). Retention times were approximately 8 and 11 minutes for the T-705M1 metabolite and T-705, respectively. A standard curve was run using T-705 and T-705M1 at 1, 4, 10, 40, and 100 µg/ml. Using this curve, peak areas of samples were converted to µg/ml plasma concentrations.

### Statistical analysis

Kaplan-Meier survival plots were generated using the Prism software package (GraphPad Software, San Diego, CA). Survival curves were compared using the log-rank test. The Mann-Whitney test (two-tailed) was performed to analyze the differences in mean virus titers and serum ALT levels.

## Results

### Virologic and disease parameter values during the course of PICV infection

**Figure 1:**
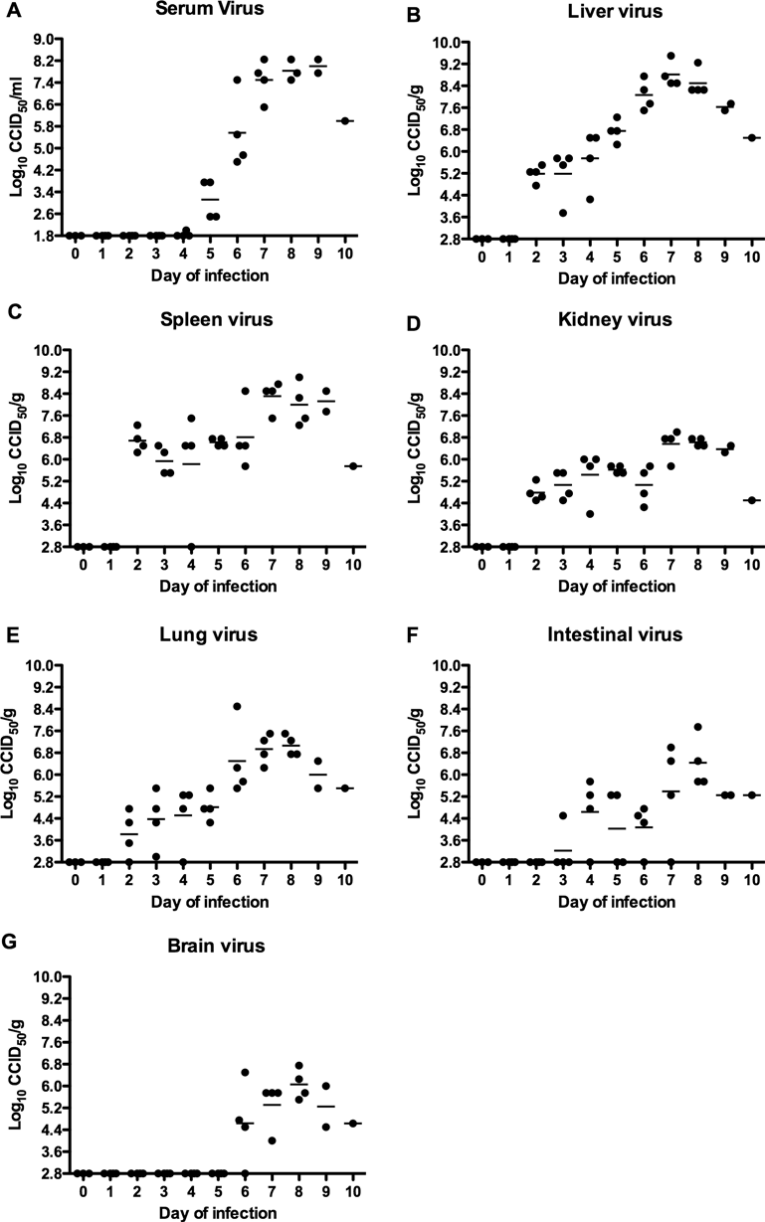
Serum and tissue virus burden during the course of PICV infection in hamsters. All daily sacrifice groups had 4 hamsters/group except for the day-0 control group, which had 3 animals. Due to death prior to time of sacrifice, tissue and serum samples could only be obtained for 2 and 1 hamsters for the day 9 and 10 groups, respectively. The serum sample was unobtainable from 1 of the day-8 hamsters due to death just prior to sacrifice. (A) Serum, (B) liver, (C) spleen, (D) kidney, (E) lung, (F) intestinal, and (G) brain virus titers were determined by infectious virus cell culture assay. Data points represent viral loads for individual hamsters with the group mean represented by the respective horizontal bar.

It is important to assess animal efficacy data in the context of the natural history of disease. To do this, landmark events indicative of disease must be established. The data presented in Figure 1 indicate the times at which viral burden becomes apparent and when peak levels are reached during PICV infection in adult hamsters. Systemic viral burden was not apparent until the fifth day of the infection (Figure 1A). By day 7, near maximal titers of 7.5–8.5 log_10_ were seen in most hamsters. In contrast, modest levels of liver virus were detectable as early as day 2, gradually increasing until reaching peak levels of >8.5 log_10_ by day 7 (Figure 1B). Modest splenic viral loads were also detectable by day 2 and fluctuated only slightly through day 6 of the infection, with mean titers ranging from 5.8 to 6.8 log_10_ (Figure 1C). Peak spleen viral titers of ∼8 log_10_ were seen from days 7–9. Our studies confirmed the conclusions of others that liver and spleen are the major target organs in hamsters infected with PICV [21].

A previous study based on PICV infection of weanling age hamsters reported high kidney, lung, and brain viral titers [17]. In this study, we examined these same tissues, as well as the intestines, for viral burden. Kidney viral titers showed a similar trend to that seen with spleen virus: virus was detectable on day 2, with only minimal fluctuation through day 6 of the infection, before spiking to peak levels on days 7–9 (∼6.5 log_10_; Figure 1D). Despite the similar trend, kidney virus load was generally 1–2 log_10_ less compared to that of spleen. Lung virus was detectable by day 2 of infection and viral loads increased daily to ∼7 log_10_ by days 7 and 8 (Figure 1E). The intestines appeared to be more refractory to infection as only 1 of 4 animals had detectable virus on day 3 and at least one hamster in each daily sacrifice group through the first 7 days had undetectable levels of intestinal virus (Figure 1F). All sacrificed animals from the day-8, -9 and -10 groups presented with modest intestinal viral burdens, with a mean peak titer of ∼6.5 log_10_ on day 8. As with other tissues, Smee and colleagues found >8 log_10_ of infectious virus in the brains of PICV-infected weanling animals and viral replication was detected as early as day 2 [17]. In this study, virus was not detected in the brain until day 6, with the peak titer of ∼6 log_10_ detected on day 8 of the infection (Figure 1F). The observed difference between the studies is most likely due to the fact that Smee et al. [17] used an infectious PICV inoculum that was 500 times (1,000 PFU) the amount used in the present study.

**Figure 2:**
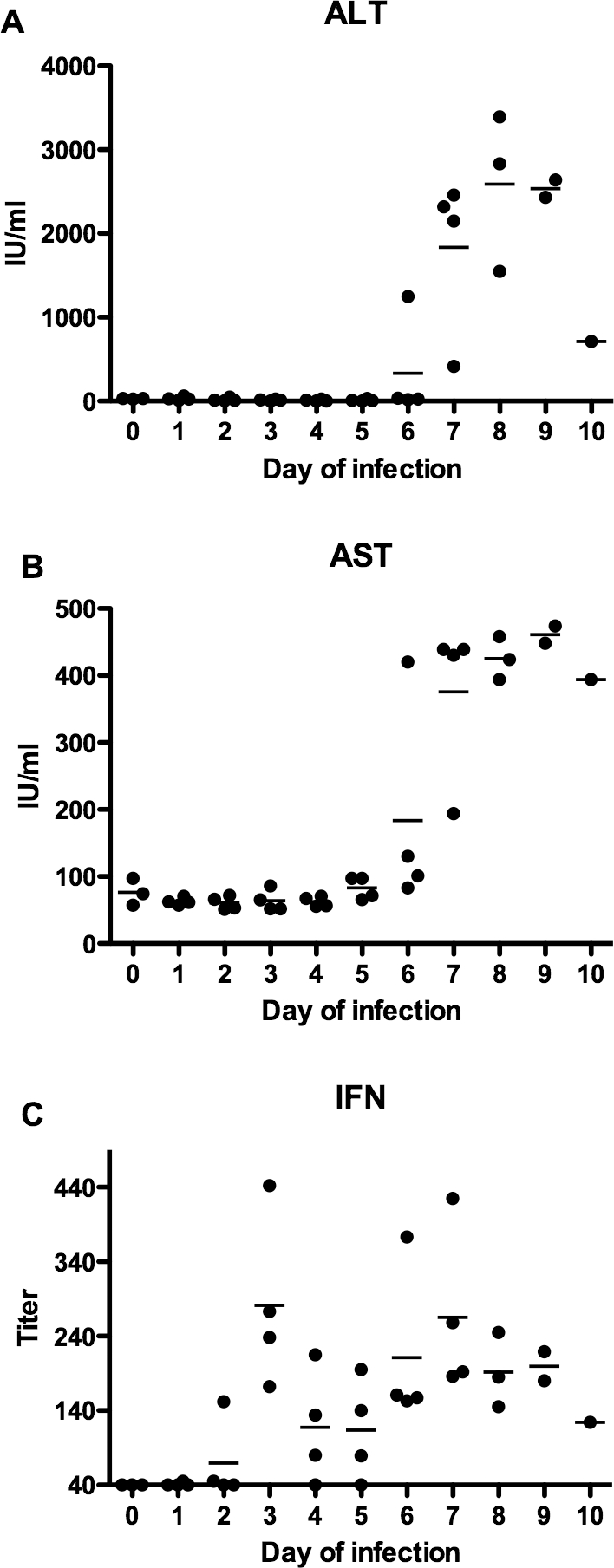
Systemic ALT, AST and type I IFN levels during the course of PICV infection in hamsters. Serum samples assayed for viral burden, as described for Figure 1, were also assayed for (A) ALT, (B) AST, and (C) type I IFN. Data points represent levels for individual hamsters with the group mean represented by the respective horizontal bar.

In addition to examining the kinetics of infectious virus during the course of PICV infection, several clinical markers and type I IFN induction were also assessed. Baseline amounts of ALT and AST were found through the first 5 days of infection, with a single hamster having elevated readings for both enzymes on day 6 (Figure 2A, B). Although markedly elevated in most animals on day 7, ALT and AST generally reached peak levels on days 8 and 9. The single surviving animal on day 10 of the infection presented with relatively lower ALT and high AST. Type I IFN was also assessed using a bioassay based on the inhibition of CPE induced by VSV infection of BHK-21 cells treated with dilutions of serum samples collected on the various days of infection. A wide-range of IFN titers was observed throughout most of the experiment without a clear pattern (Figure 2C). IFN activity was detected as early as day 2 in one of the infected animals and the highest titers were collectively seen on day 3.

### Delayed treatment of PICV-infected hamsters with T-705 and ribavirin

We have previously demonstrated that for early stage treatment of a highly lethal PICV challenge, the lowest effective doses of T-705 and ribavirin were 50 and 20 mg/kg/day, respectively [8]. Since our objective in the present study was to initiate treatment later during the course of infection when higher viral burdens are encountered, the T-705 and ribavirin doses were increased to 100 mg/kg/day and 40 mg/kg/day, respectively, as a means to compare the antiviral activity of the drugs based on twice their previous lowest effective doses. Treatments were initiated on days 4, 5 or 6, based on the longitudinal analysis of the PICV model described above (Figures 1 and 2), and a small preliminary study (data not shown) wherein treatment initiated by day 4 completely prevented disease and inhibited viral replication in a small number of animals, with some evidence of protection when initiated on day 6.

**Figure 3:**
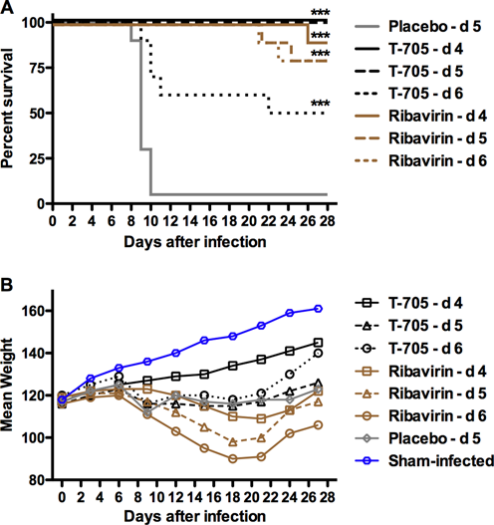
Effect of delayed T-705 and ribavirin therapy on PICV infection outcome in hamsters. Drugs were given orally twice per day for 7 days. Treatments were initiated on day 4, 5 or 6. T-705 was administered as a daily dose of 100 mg/kg. Ribavirin was dosed at 40 mg/kg/day. Ten animals per group (20 for the placebo) were observed daily for 4 weeks for (A) survival and every third day for (B) mean group weight determination of survivors. ***P<0.001 compared to 0.4% CMC placebo-treated hamsters.

Hamsters treated orally with 100 mg/kg/day of T-705 starting 4 or 5 days post challenge with PICV survived an infectious inoculum that killed 95% of animals given 0.4% CMC placebo (Figure 3A). As seen previously, protection waned if T-705 therapy was initiated on day 6; however, in this experiment, the reduced efficacy observed (50% survival) was highly significant compared to the placebo control (P<0.001). All ribavirin treatments were effective protecting >80–90% of infected animals from death. Remarkably, many of the surviving animals treated with ribavirin were generally in poor condition, smaller and dehydrated compared to surviving animals that received T-705 or placebo. This observation was borne out in the analysis of mean group weights that were assessed every 3 days starting on day 0 (Figure 3B). In all cases, the animals treated with ribavirin presented with the most dramatic weight loss that was never fully recovered in the day-6 treatment group. Although the mean weights of the animals treated with T-705 were also less than seen in the sham-infected control animals, all T-705 groups were heavier (Figure 3B) and in better condition compared to the ribavirin groups.

**Figure 4:**
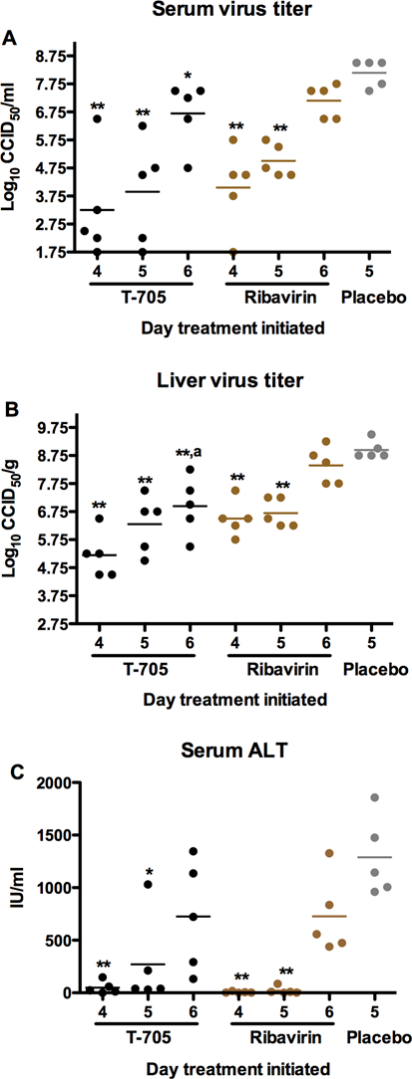
Reduction of viral load and liver disease in PICV-challenged hamsters following delayed treatment with T-705 and ribavirin. Five hamsters per group were treated as described in Figure 3, and sacrificed on day 7 of the infection to assess (A) systemic and (B) hepatic viral burden, and liver disease as determined by (C) ALT measurement. Data points represent values for individual animals and mean virus titers and ALT levels for each group are represented by horizontal lines. *P<0.05; **P<0.01 compared to 0.4% CMC placebo-treated hamsters. ^a^ P<0.05 compared to hamsters receiving ribavirin treatment starting on the same day.

In respective groups of parallel infected and treated hamsters sacrificed on day 7 of PICV infection to measure virological and liver disease parameters, all treatment groups, with the exception of the day-6 ribavirin group, had significantly reduced serum and liver viral titers, with T-705 producing the most dramatic decreases (Figure 4A, B). As treatments were delayed out to day 6, viral burden increased. This pattern was also evident in the analysis of serum ALT levels with the early (day-4 initiation) treatment groups generally having the lowest values (Figure 4C).

A combination study wherein PICV-challenged hamsters were treated with suboptimal doses of T-705 and ribavirin starting on day 5 of infection was conducted to investigate whether such a strategy could reduce the effective dosage of ribavirin in order to limit its toxicity. Unfortunately, we were not able to achieve the 100% survival obtained with daily doses of 100 mg/kg of T-705 or 60 mg/kg of ribavirin using combinations of smaller doses of each drug (Table 1). Serum viral titer and ALT data were suggestive of an additive effect, with a weak indication of improved activity in the context of liver viral load reduction. The results from this initial study investigating combination therapy did not warrant further exploration of synergy between the two drugs.

### Treatment of late stage infection with equitoxic doses of T-705 and ribavirin

**Figure 5:**
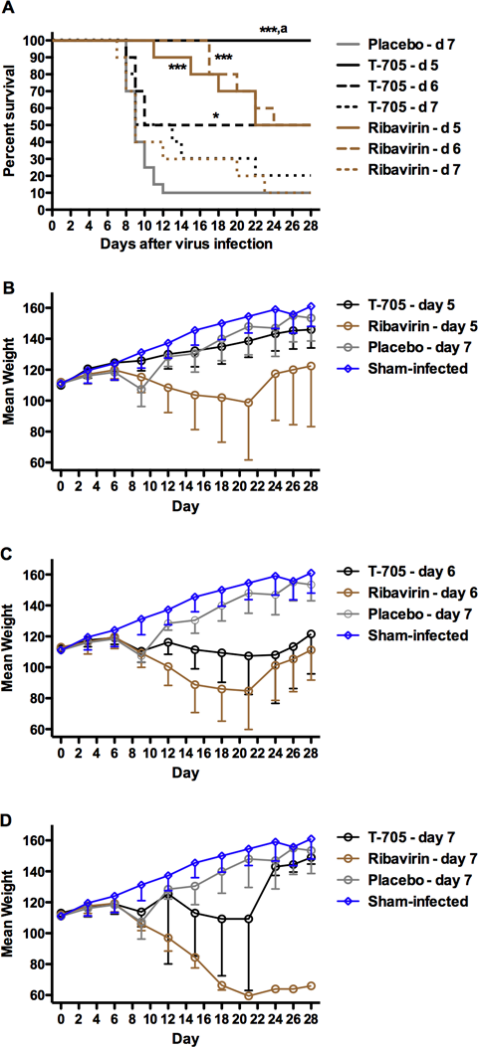
Effect of delayed treatment with equitoxic doses of T-705 and ribavirin on PICV infection in hamsters. Drugs were given orally twice per day for 7 days starting on day 5, 6 or 7 for the drug-treated groups and day 7 for the placebo group. T-705 was dosed at 160 mg/kg/day on the first day and 80 mg/kg/day thereafter. Ribavirin was given at 22 mg/kg/day on the first day and 11 mg/kg/day thereafter. Hamsters were observed 28 days for (A) survival and weights and standard deviations of survivors are shown for treatments initiated on day (B) 5, (C) 6 or (D) 7. The 0.4% CMC-treated placebo and sham-infected, untreated groups are included in B–D for comparison. For the survival analysis, *P<0.05; ***P<0.001 compared to placebo-treated hamsters by log-rank test. ^a^ P<0.05 compared to hamsters receiving ribavirin treatment starting on the same day.

Although ribavirin treatment initiated 6 days after challenge with PICV protected 80% of the animals against death, the mean weight of the survivors was ∼25% less than the 5 animals that survived the equivalent T-705 treatment. Thus, although ribavirin appeared to be more effective in the context of survival, the animals were clearly not as well off as the vast majority of the survivors from the T-705 treated groups, suggesting underlying toxicity. Consequently, we next used approximate equitoxic doses of T-705 and ribavirin to treat PICV infection. Treatments were initiated on days 5, 6 or 7 with an initial loading dose that is approximately 10-fold less than the LD_50_. Because the LD_50_ of T-705 in hamsters is >1,500 mg/kg/day, for a 7-day twice a day regimen [8], we assigned 1,600 mg/kg/day as the LD_50_, which is likely an underestimate. The LD_50_ for ribavirin in hamsters treated by the same route and schedule is 220 mg/kg/day [8]. Therefore, T-705 was dosed at 160 mg/kg/day on the first day and 80 mg/kg/day thereafter, and ribavirin was given at 22 mg/kg/day on the first day and 11 mg/kg/day afterwards. When treatment was initiated on day 5 of the infection, 100% of the hamsters receiving the T-705 regimen were protected (Figure 5A). Compared to the ribavirin treatment group which had 50% mortality, T-705 treatment significantly improved survival outcome at the equitoxic doses tested when initiated 5 days after infection. Notably, the average weights of the ribavirin-treated animals recovering from PICV challenge were less than those of the survivors from the other experimental groups (Figure 5B).

When treatment was delayed until day 6 of the infection, both ribavirin and T-705 protected 50% of the animals, but ribavirin greatly extended the time-to-death (Figure 5A). Average weights were comparable between the two drug treatment groups late in the experiment when the surviving animals were recovering from disease (Figure 5C). Treatments withheld until day 7 were only slightly beneficial, in that death was delayed in a few of the animals (Figure 5A). Remarkable weight loss was observed for the ribavirin-treated animals and the sole survivor was grossly underweight (Figure 5D) and ataxic on day 28. Only one other animal (in the day-5 ribavirin treatment-initiation group) presented with a similar condition at the conclusion of the study. These two animals, as well as a large cohort of survivors from the other groups, were examined for brain and serum viral titers on day 28. Detectable amounts of virus were not present in any of the samples using the infectious virus assay employed (data not shown), which had a limit of detection of 2.8 log_10_ CCID_50_/g of tissue or 1.8 log_10_ CCID_50_/ml of serum.

**Figure 6:**
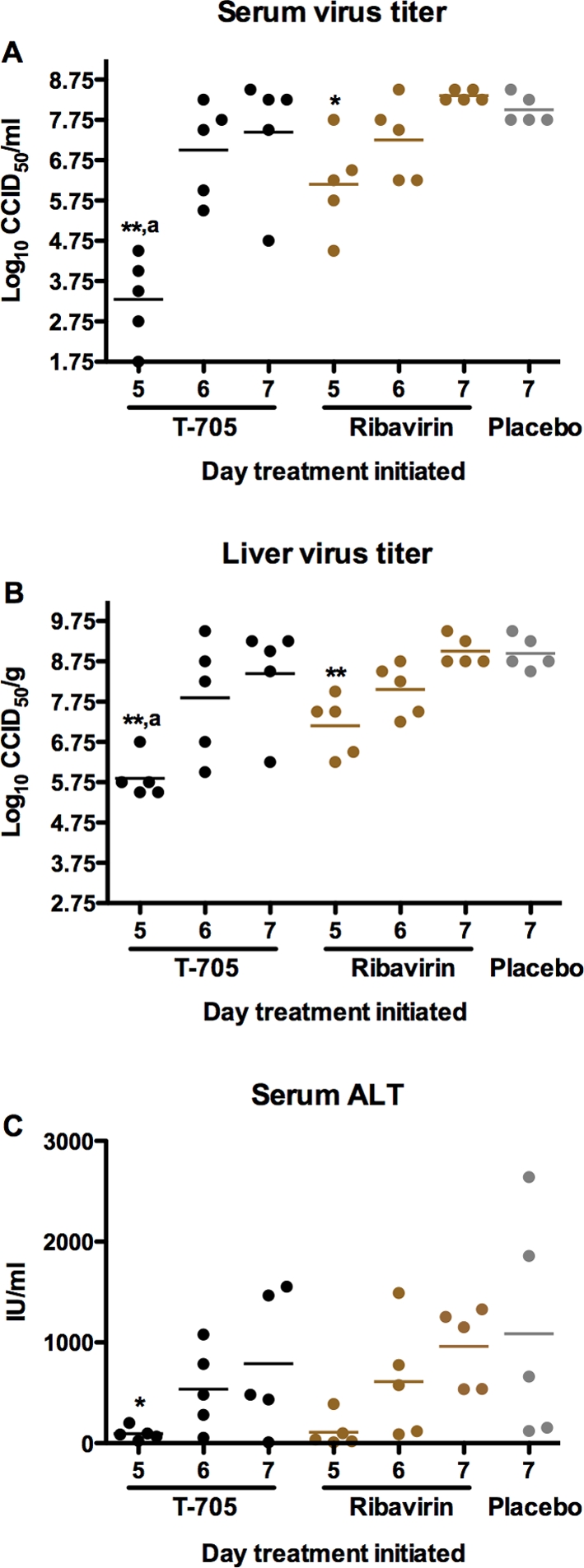
Reduction of viral load and liver disease in PICV-challenged hamsters following delayed treatment with equitoxic doses of T-705 and ribavirin. Five hamsters per group were treated as described in Figure 5, and sacrificed on day 7 of the infection to assess (A) systemic and (B) hepatic viral burden, and (C) ALT. The day-7 treatment initiation group only received the morning dose, with animals sacrificed 3-4 h after treatment. Data points represent values for individual animals and mean virus titers and ALT levels for each group are represented by horizontal lines. *P<0.05; **P<0.01 compared to 0.4% CMC placebo-treated hamsters. ^a^ P<0.05 compared to hamsters receiving ribavirin treatment starting on the same day.

In the evaluation of viral burden in hamsters sacrificed on day 7, the T-705 treatment initiated on day 5 significantly reduced both systemic and liver tissue viral loads compared to the placebo group, with >5.5 and 3 log_10_ reductions observed, respectively (Figure 6A, B). The day-5 ribavirin treatment also significantly reduced viral burden, but to a lesser degree. All other drug treatment groups had only minimal, if any, impact on viral burden. Only the day-5 T-705 group significantly prevented liver damage as determined by measurable ALT activity found in the serum (Figure 6C). Notably, the day-5 ribavirin group also presented with lower ALT values; however, this may be a reflection of the extended survival time seen in the animals that eventually perished.

### High-dose T-705 treatment of late stage PICV virus infection

**Figure 7:**
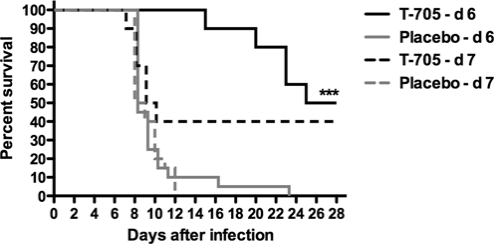
Survival outcome following high-dose treatment of advanced PICV infection in hamsters. T-705 or placebo was given orally twice per day for 8 days starting on day 6 or 7. T-705 was dosed at 320 mg/kg/day on the first day and 100 mg/kg/day thereafter. Hamsters were observed 28 days for survival. ***P<0.001 compared to respective placebo-treated hamsters by log-rank test.

It is possible that a higher initial dose of T-705 may be more effective in reducing the massive viral burden that is present in infected hamsters on day 7. A more aggressive dosing regimen may result in significant protection of challenged animals when initiating treatment at such an advanced stage. Thus, an experiment was designed wherein a loading dose of 320 mg/kg would be used for the first day, followed by 7 days of treatment with 100 mg/kg/day. By doubling the dose and extending the duration of treatment, we were able to protect a significant (Fisher's exact two-tailed test, *P* = 0.0077; log-rank test, *P* = 0.0577) number of hamsters when initiating treatment on day 7 (Figure 7). Interestingly, the 40% survival observed in the day-7 treatment group was similar to the 50% survival seen when treatment was initiated a day earlier. Notably, however, the precipitous survival curve for the day-7 group closely resembled those of the placebo-treated groups, whereas those animals that started receiving T-705 a day earlier survived 5–15 days longer (Figure 7).

**Figure 8:**
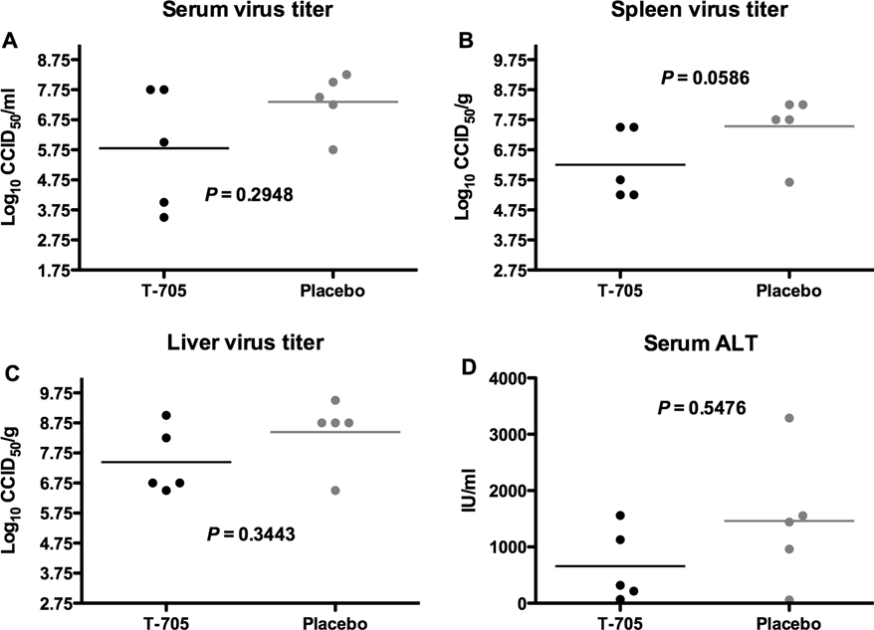
Effect of delayed high-dose T-705 therapy on viral burden and liver disease in PICV-challenged hamsters. Five hamsters per group for the treatment groups initiated on day 6 were treated as described in Figure 7, and sacrificed on day 7 of the infection to assess (A) systemic, (B) splenic, and (C) hepatic viral loads, and (D) ALT. Data points represent values for individual animals and mean virus titers and ALT levels for each group are represented by horizontal lines.

As in the previous experiments, parallel infected and treated hamsters were sacrificed on day 7 of PICV infection to examine the impact of treatment on viral load and liver disease. In addition to serum and liver viral burden, titers were also assessed for spleen tissue in this experiment, as it is a major target organ for PICV infection in hamsters [21]. Although titers were not significantly reduced by T-705 treatment initiated the day prior to sacrifice, mean viral loads were generally lower in the animals that received T-705 (Figure 8A–C). Mean serum ALT concentrations were also lower in the T-705 treated hamsters, with 80% of the placebo-treated animals having serum ALT >1,000, compared to only 40% for the T-705 group (Figure 8D).

### Reduced plasma concentrations of T-705 during late stage infection

**Figure 9:**
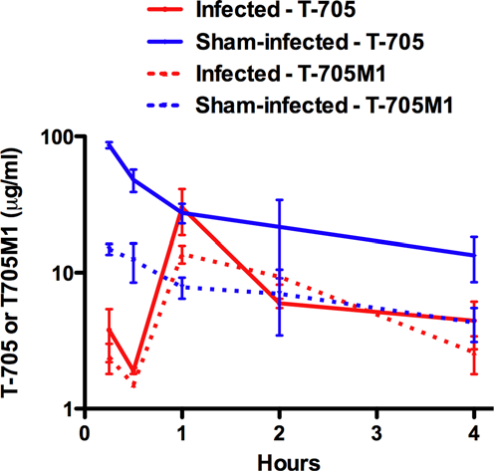
Reduced systemic absorption of orally administered T-705 in PICV-infected hamsters. Infected or sham-infected animals were treated with 50 mg/kg of T-705 on day 7 of infection and plasma samples were taken from animals sacrificed at 0.25, 0.5, 1, 2 or 4 h after treatment. Samples were processed and analyzed by HPLC for T-705 and its primary metabolite, T-705M1, as described in the methods section. Data represent the mean and standard error of the mean from 3 hamsters (n = 3) per time point.

It is conceivable that reduced plasma concentrations of orally administered T-705 may result as a consequence of altered gastric absorption, tissue distribution, and/or elimination of the drug in sick animals. Lower concentrations of T-705 would limit the amount of available drug to combat the high PICV loads present upon initiation of treatment on day 6 and 7. This possibility was investigated by HPLC analysis of plasma taken from PICV-infected and sham-infected animals given oral T-705 on day 7 of infection. Plasma T-705 and T-705M1 concentrations from infected and uninfected animals are compared in Figure 9. T-705 was quickly absorbed into the circulation of sham-infected animals achieving plasma concentrations comparable with previously conducted PK studies in hamsters and mice (Y. Furuta et al., unpublished data). In contrast, T-705 concentrations in PICV-infected animals demonstrated vastly different kinetics, with dramatically lower concentrations seen at the early time points, followed by a peak at the 1 h time point, with a sharp decline at 2 h (Figure 9). Notably, the dead-end metabolite, T-705M1, was more prominently found in the infected animals with much higher ratios of T-705M1:T-705 being observed compared to the sham-infected hamsters. It is possible that a defect in the elimination or distribution of the T-705M1 metabolite may be causing this shift.

## Discussion

Here, we describe effective late stage treatment of a severe arenaviral infection that serves as a model of arenaviral HF with the novel pyrazine derivative, T-705. In addition to the main objective of determining whether T-705 therapy could be effective when delaying the onset of treatment, the efficacy of T-705 was also compared to that of ribavirin, as it is currently the only antiviral considered for use for the treatment of arenaviral HFs [7], [22]. Notably, the ribavirin-treated animals generally lost considerably more weight than those receiving T-705 and survivors recovered more slowly as indicated by the lowest levels of weight recuperation at the conclusion of the studies. Conceivably, residual toxic effects due to the treatment may have weakened the animals while combating the infection, thus attenuating the recovery process.

In the first efficacy experiment, the mean day of death for the T-705-treated animals in the day-6 initiation group (12.4±5.4) was 3 days later than in the placebo group (9.2±0.6), while the animals treated with ribavirin did not begin to die until the twentieth day of the infection, well after the treatments had ceased. It is possible that the viral infection may have rebounded in some of the ribavirin-treated animals following the end of treatment. Extending the duration of treatment may have resulted in increased protection by ribavirin. Nevertheless, there is uncertainty in this regard considering the dramatic weight loss and toxicity seen during the 7-day treatment regimen.

It is important to note that because of its lower toxicity, we were not able to determine an LD_50_ for T-705, as a 1,500 mg/kg/day dosing regimen only caused temporary weight loss [8]. Therefore, the estimated LD_50_ used to determine the T-705 dose used in the second study comparing administration of equitoxic amounts of drug is likely an underestimation of the actual T-705 toxicity. With that said, T-705 therapy was more effective than ribavirin at protecting animals from death and limiting viral replication and liver disease when treatments were initiated on day 5 of PICV infection. Interestingly, in the equitoxic dosing study, as well as the initial evaluation, ribavirin was as effective or better in the context of survival when treatment was delayed until day 6. This trend, however, was not observed when treatment was begun on day 7, as only a slight hint of protection was seen with either drug.

Currently, ribavirin is the only drug that has demonstrated efficacy in humans when administered i.v. within 6 days from the onset of illness [6]. The idea that T-705 and ribavirin impart their antiviral activities in different ways suggests that they may act synergistically in treating arenaviral infections and ultimately provide the most robust treatment modality. Unfortunately, there was no evidence of synergy with the combined therapy employing a limited amount of suboptimal doses of ribavirin and T-705 with treatments initiated 5 days after PICV challenge. Studies investigating the potential combination of T-705 and ribavirin in nonhuman primate models of arenaviral HF may be warranted.

During the temporal analysis of viral burden in hamster tissues, we found that PICV is initially detectable in the brain by day 6 of infection in most animals. Lassa virus does not generally cause neurologic disease, although the virus has been isolated from CNS fluid [23]. In contrast, infections with Junin virus can produce encephalitic disease [24], [25], [26]. An interesting observation resulting from the efficacy studies was that several of the smaller ribavirin-treated survivors appeared to be very uncoordinated with mild ataxia during the end stages of the experiments, suggesting neurologic disease. To further investigate this possibility, the majority of the surviving hamsters from the second experiment, where equitoxic doses of T-705 and ribavirin were compared, were assayed for the presence of infectious virus in the brain on day 28. No detectable virus was found in the brains or serum of any of the animals sampled. The lack of measurable virus in the brain of hamsters presenting with mild ataxia and tremors observed only in two of the survivors that had been treated with ribavirin suggests that signs of neurologic disease may be due to immunopathology, secondary effects of other system damage, or viral load less than the limit of measure. Additional investigation into this matter may be warranted and would require histopathological and immunohistochemical analysis of brain and spinal cord tissue.

Remarkably, significant efficacy was observed with high-dose (320 mg/kg/day loading, 100 mg/kg/day thereafter) oral T-705 therapy initiated as late as day 7, a stage when peak systemic and tissue viral titers are evident in most tissue compartments. In the majority of cases, animals sacrificed on day 7 had maximal viral burden and AST concentrations, which is consistent with the greatest levels of mortality observed in groups wherein treatments were initiated at such advanced stages of disease. Although we were able to extend the time to death in animals treated with high-dose T-705 starting on day 6 of PICV infection, 50% of the hamsters eventually succumbed. Close examination of viral burden and ALT concentrations in the animals that began treatment on day 6 suggests that there may be certain thresholds for these parameters that once reached are strong prognosticators of mortality, despite therapeutic intervention. Indeed, in human Lassa fever cases, viremia and serum AST levels are strong predictive factors of disease outcome [6]. Alternatively, the dramatically lower T-705 plasma concentrations observed in hamsters treated on the seventh day of infection may also result in animals treated on day 6. This reduced amount of systemic T-705 may be sub-optimal to combat the advanced stage arenaviral infection.

Considering that panorganotropic arenaviral infections may diminish kidney function, in-depth analysis of the effects of PICV infection on T-705 PK, pharmacodynamics, biodistribution, and metabolism, all of which may limit the therapeutic timeframe, are warranted. Moreover, comprehensive longitudinal studies to clearly define the relationship between T-705 efficacy and p.o. absorption are needed. Because reduced plasma concentrations of T-705 in infected animals following oral T-705 administration was so prominent on day 7, it may be possible to improve upon the current levels of protection observed by administering T-705 i.v. During severe disease, patients are often too sick to swallow oral medications, and may be too volume-depleted to absorb and distribute a drug that arrives in the stomach. Consequently, i.v. administration would likely be the standard of care. In hamsters, however, it is not currently possible to treat i.v., as there is currently no feasible way to effectively administer drug treatments multiple times per day for an extended period of time. We are currently exploring retro orbital i.v. injection as a potential means of evaluating T-705 efficacy at times when gastric absorption is depleted and increased vascular permeability would likely hinder drug absorption following i.p., intramuscular, or subcutaneous delivery. In addition, studies are being designed to evaluate T-705 in cell culture and animal models based on infection with authentic highly pathogenic arenaviruses such as Lassa and Junin viruses.

The mechanism of action of T-705 against arenaviruses is not known. Against influenza A virus (IAV), time-of-addition studies with T-705 showed that the drug targets an early to middle stage of the viral replication cycle that involves viral polymerase activity. In competitive inhibition studies, the anti-IAV activity of T-705 was reduced by the addition of purines and purine nucleosides indicating that T-705 functions as a purine analog. Further, T-705RTP inhibited IAV RNA-dependent RNA-polymerase (RdRp) in a dose-dependent and GTP-competitive manner. Importantly, T-705 was not found to inhibit cellular DNA and RNA synthesis, which are processes inhibited by ribavirin and other inhibitors of cellular inosine monophosphate dehydrogenase (IMPDH), a key enzyme in the purine salvage pathway [27]. Studies are currently underway to determine whether selective inhibition of New World arenaviral RdRp will be the principal mechanism of T-705 action. The reduced toxicity of T-705 and its broad-spectrum activity against a number of RNA viruses may result from its ability to target RdRp domains, since they are not present in the host and are conserved among RNA viruses.
